# Precise Control of Micropipette Flow Rate for Fluorescence Imaging in In Vivo Micromanipulation

**DOI:** 10.3390/s25216647

**Published:** 2025-10-30

**Authors:** Ruimin Li, Shaojie Fu, Zijian Guo, Jinyu Qiu, Yuzhu Liu, Mengya Liu, Qili Zhao, Xin Zhao

**Affiliations:** 1 Institute of Robotics and Automatic Information System, Tianjin Key Laboratory of Intelligent Robotics, Nankai University, Tianjin 300350, China; lrumin@mail.nankai.edu.cn (R.L.); fushaojie0525@163.com (S.F.); 2120230544@mail.nankai.edu.cn (Z.G.); qiujinyu@mail.nankai.edu.cn (J.Q.); liuyuzhu@mail.nankai.edu.cn (Y.L.); 1120240300@mail.nankai.edu.cn (M.L.); zhaoqili@nankai.edu.cn (Q.Z.); 2 Institute of Intelligence Technology and Robotic Systems, Shenzhen Research Institute of Nankai University, Shenzhen 518083, China; 3 National Key Laboratory of Intelligent Tracking and Forecasting for Infectious Diseases, Nankai University, Tianjin 300350, China

**Keywords:** micropipette flow regulation, flow-rate calibration, fluorescence imaging, in vivo manipulation, fluorescent dye injection

## Abstract

Precise regulation of micropipette outlet flow is critical for fluorescence imaging in vivo micromanipulations. In such procedures, a micropipette with a micro-sized opening is driven by gas pressure to deliver internal solution into the in vivo environment. The outlet flow rate needs to be precisely regulated to ensure a uniform and stable fluorescence distribution. However, conventional manual pressure injection methods face inherent limitations, including insufficient precision and poor reproducibility. Existing commercial microinjection systems lack a quantitative relationship between pressure and flow rate. And existing calibration methods in the field of microfluidics suffer from a limited flow-rate measurement resolution, constraining the establishment of a precise pressure–flow quantitative relationship. To address these challenges, we developed a closed-loop pressure regulation system with 1 Pa-level control resolution and established a quantitative calibration of the pressure–flow relationship using a droplet-based method. The calibration revealed a linear relationship with a mean pressure–flow gain of 4.846 $\times \;10^{-17}m^{3}\cdot s^{-1}\cdot {Pa}^{-1}$ (R^2^ > 0.99). Validation results demonstrated that the system achieved the target outlet flow rate with a flow control error less than 10 fL/s. Finally, the application results in brain-slice environment confirmed its capability to maintain stable fluorescence imaging, with fluorescence intensity fluctuations around 1.3%. These results demonstrated that the proposed approach provides stable, precise, and reproducible flow regulation under physiologically relevant conditions, thereby offering a valuable tool for in vivo micromanipulation and detection.

## 1. Introduction

Precise regulation of outlet flow of a micropipette is essential for in vivo micromanipulations and detections, including in vivo microinjection [1,2,3], fluorescence imaging [4,5], and cellular content extraction [6]. Taking in vivo fluorescence imaging as an example, it is achieved by micropipette to inject cell-invasive fluorescent dyes to light the surrounding extracellular space and shadow the cells [3]. In the above process, outlet flow rate of the fluorescent solution should be matched with the diffusion dynamics of the dye in the tissue microenvironment; only when injection and diffusion are balanced can a stable fluorescence distribution be established [7]. Fluctuations in flow can lead to unstable background signals, or imaging artifacts, which in turn compromise the accuracy of cell localization. Similar requirements exist in other in vivo micromanipulations. In both cellular drug injection and cellular content extraction, an unstable outlet flow of operation micropipette may lead to unpredictable sampling volumes, thereby reducing reproducibility and potentially damaging cells [8]. Across these applications, ensuring a stable and reproducible outlet flow rate of operation micropipette is therefore critical not only for experimental precision but also for biological safety.

In these flow rate control applications, gas is commonly employed as the driving force, since liquid-driven systems require more complex structural designs and carry a higher risk of contamination which is detrimental to in vivo environment [9]. However, the compressibility of gas introduces significant challenges for accurate flow regulation, as variations in applied pressure do not translate linearly into stable outlet flow. In addition, there are tissue decries in complex in vivo environment, affecting solution stably flow out from the micropipette opening. Thus, it is a challenging task to achieve precise and stable injection flow in living environment.

A common micropipette flow regulation approach is to manually adjust the injection pressure inside the micropipette. In this case, the operator must continuously observe the injected outcome and adjust the pressure in real time, which is time-consuming and labor-intensive. Furthermore, manual operation suffers from poor repeatability and limited precision [10,11,12,13]. To overcome these limitations, various commercial microinjection systems have been developed [14,15,16,17]. Most commercial devices allow the operator to set or monitor injection pressure. Nevertheless, they typically operate in an open-loop manner, where the applied pressure is constant, not involving actively regulating. As a result, the outlet flow often becomes unstable and uncontrollable, especially under dynamic conditions or when the micropipette tip encounters partial blockage. More advanced systems have attempted closed-loop regulation of injection pressure for specific applications such as patch clamp recordings [18]. They established the pressure regulating system, which employed the PID algorithm to achieve the pressure control. Although these approaches represent significant progress, they generally lack a quantitative characterization of the relationship between injection pressure and outlet flow rate. Without such calibration, achieving reliable and reproducible flow control remains a major challenge.

To construct such a relationship, both pressure and flow rate must be measured simultaneously. However, to achieve precise outlet flow rate control, it is essential to measure the flow rate at the femtoliter-per-second (fL/s) scale, which remains highly challenging. In previous studies [19], the outlet flow rate of a nanopipette was determined from the displacement of a gas–liquid meniscus, achieving precision on the order of tens of picoliters per second (pL/s). Gravimetric methods, which estimate flow rate from the time-dependent change in mass, have also been explored [20], but typically provide only milliliter-per-minute (mL/min) resolution. Overall, despite substantial progress, existing calibration approaches still lack the resolution and temporal sensitivity required to quantitatively and predictively link injection pressure with flow rate at the femtoliter scale. Furthermore, the numerical simulations [21] can study the relationship between injection pressure and outlet flow rate, experimental calibration under real, complex environments remains indispensable for ensuring accurate flow control in biological applications.

In the microfluidic systems, recent works have achieved microliter-per-minute (uL/min) and (nL/min) resolution [22,23,24,25]. Such platforms achieve excellent reproducibility and have contributed significantly to precise manipulation of micro-scale fluids in chip-based experiments. While most of these studies are designed for in vitro, enclosed-channel environments, where flow is confined within microchannels under well-controlled boundary conditions. They are not suitable for direct insertion into brain tissue or for dynamic flow control in in vivo environments.

In this study, we presented a novel closed-loop system for precise regulation of micropipette outlet flow. We first developed a multi-channel pressure regulation unit with three independent channels, offering fine (1 Pa resolution) outputs for both positive and negative pressure ranging from −100 hPa to 100 hPa and atmospheric pressure, thereby accommodating a wide range of experimental requirements. To enable accurate dynamic pressure control, we first established the pressure–voltage relationship of the electronic pressure controllers (EPCs). Based on this model, we implemented a control strategy combining anti-integral saturation feedforward PID regulation with Kalman filtering of pressure sensor data. This approach allowed precise adjustment of the EPC input voltage and achieved stable and accurate injection pressure. Furthermore, we experimentally calibrated the quantitative pressure–flow relationship, bridging the gap between pressure control and reproducible flow regulation. For calibration, the micropipette tip was immersed into silicone oil and the injected solution formed droplets, whose volume growth was recorded under a microscope. The outlet flow rate was then calculated as the time derivative of droplet volume. Static calibration experiments revealed a linear pressure–flow relationship within the working range, with a mean gain of 4.846 $\times \;10^{-17}m^{3}\cdot s^{-1}\cdot {Pa}^{-1}$ (R^2^ > 0.99).

Combining this calibration result with the injection model, we conducted validation experiments by injecting fluorescent dye into silicone oil under controlled pressure conditions. Target flow rates were set based on the calibrated pressure–flow gain, and the measured flow rates were compared with these targets. As a result, the system achieved outlet flow regulation with a flow control error less than 10 fL/s, demonstrating that the proposed approach successfully bridges the gap between pressure control and reproducible flow regulation. Finally, application to fluorescent dye lighting in brain slices confirmed that the system enabled stable fluorescence imaging, maintaining fluorescence intensity fluctuations around 1.3%. Together, these results highlight that the proposed system provides stable, precise, and reproducible outlet flow regulation, thereby addressing a critical challenge in in vivo micromanipulation.

## 2. Materials and Methods

### 2.1. System Setup

**Figure 1:**
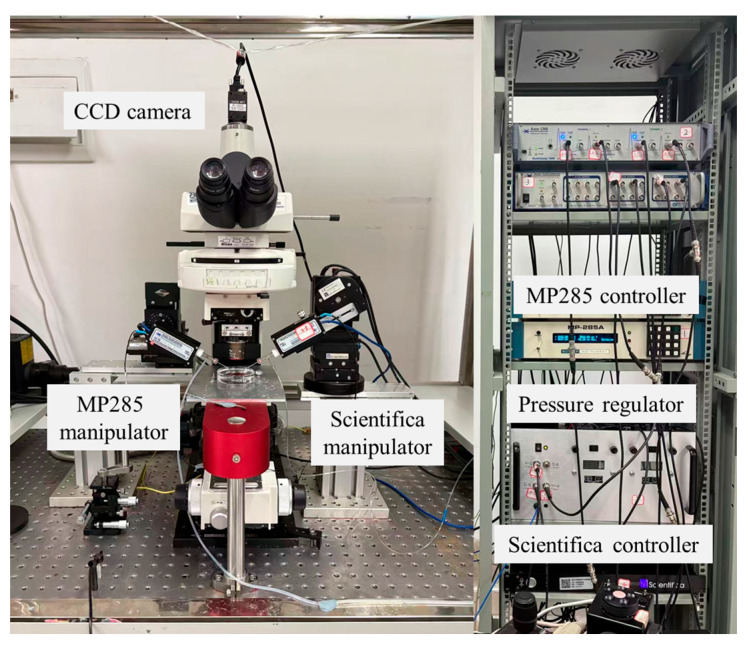
Experimental system setup used for calibration and validation. The system integrates an upright microscope, CCD imaging, motorized XYZ stage, and dual micromanipulators to enable precise measurement of micropipette outlet flow rate.

In this study, system calibration and validation were conducted using the experimental setup shown in Figure 1. An upright microscope (Eclipse FN1, Nikon, Tokyo, Japan) is used to measure the micropipette outlet flow rate by recording the droplet volume change (details in Section 2.4). Motorized XYZ stages are integrated with the microscope, enabling precise positioning of the microscopic field of view within a 2 × 2 × 2 cm^3^ working space. A CCD (IR-2000, DAGE-MTI, Michigan City, IN, USA) camera is mounted on the microscope and transfers the vision information to the computer, capturing images at up to 60 frames per second. Two micromanipulators are located on both sides of the microscope. The left micromanipulator (MP285, Sutter, Novato, CA, USA, workspace: 2 × 2 × 2 cm^3^, maximum speed: 1 mm/s) is used for mounting an injection micropipette. The right micromanipulator (S-PS-7000C, Scientifica Ltd, Uckfield, UK, workspace: 2 × 2 × 2 cm^3^, maximum speed: 4 mm/s) is used to hold an auxiliary micropipette, which assists the injection micropipette in breaking through the liquid tension and injecting into the silicone oil (details in Section 2.4).

**Figure 2:**
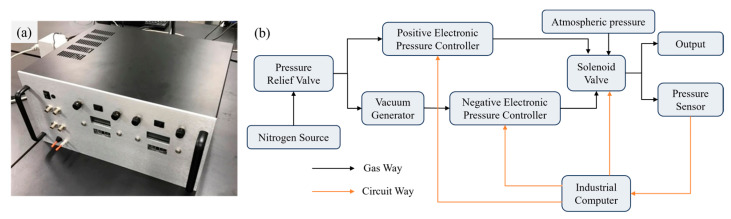
Pressure regulator for micropipette flow control. (**a**) Photograph of the assembled device. (**b**) Schematic diagram of the internal configuration, illustrating the connections among the compressed gas source, valve, pressure sensor and outlet port.

Furthermore, we developed a pressure regulating system, as illustrated in Figure 2a. The system uses high-pressure nitrogen (~0.6 MPa) from a gas cylinder as the supply source. Nitrogen is selected because it is inert, non-polluting, and safe for laboratory applications. The regulator provides three independent output channels: positive pressure, negative pressure, and atmospheric pressure.

For positive pressure output, an electronic pressure controller (900-005101-002, Parker, Cleveland, OH, USA) is employed, with its output regulated by adjusting the input control voltage. For negative pressure output, the gas supply is first passed through a vacuum generator (AVR038H, AIR-VAC, Seymour, CT, USA) and subsequently into a negative EPC (990-005201-002, Paker, Mineral Wells, TX, USA). By adjusting the input voltage of the negative EPC, the outlet negative pressure could be precisely adjusted. A solenoid valve (LHDA0533115H, The Lee Company, Westbrook, CT, USA) is employed to direct either positive, negative, or atmospheric pressure to the outlet. In addition, to ensure the input pressure remains within EPCs operational range, a pressure relief valve assembly (41795K31 and 41795K33, McMaster-Carr Supply Company, Elmhurst, IL, USA) is employed to precisely regulate the supplied nitrogen pressure.

**Figure 3:**
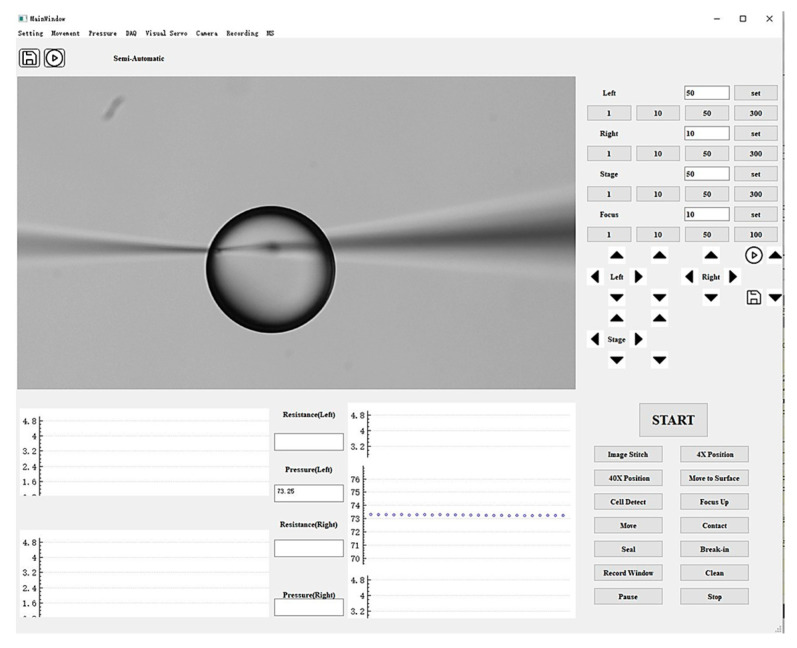
Graphical user interface of the system, consisting of a functional control panel, image display panel, and data presentation panel.

A graphical user interface (GUI) was developed using Qt to integrate the closed-loop pressure control algorithm, EPC, solenoid valves, pressure sensor, and manipulator controller, as shown in Figure 3. This interface enables the operator to adjust injection pressure, monitor real-time pressure values, and control micropipette motion. The GUI consists of three modules: a functional panel, an image display panel, and a data presentation panel. The functional panel contains control buttons for regulating injection pressure, manipulating micropipette movement, and adjusting the microscopic field of view. The image display panel provides a live view of the microscopic field. The data presentation panel plots real-time traces of injection pressure over time.

At this stage, the pressure regulator operated in open-loop mode, meaning the output pressure could not yet be stabilized or corrected against disturbances. To enable closed-loop regulation, we analyzed and modeled the relationship between the input voltage of the EPCs and the corresponding output pressures (Section 2.2), which provided the basis for implementing feedback control (details in Section 2.3).

### 2.2. Analysis and Modeling of EPC Pressure–Voltage Relationship

**Figure 4:**
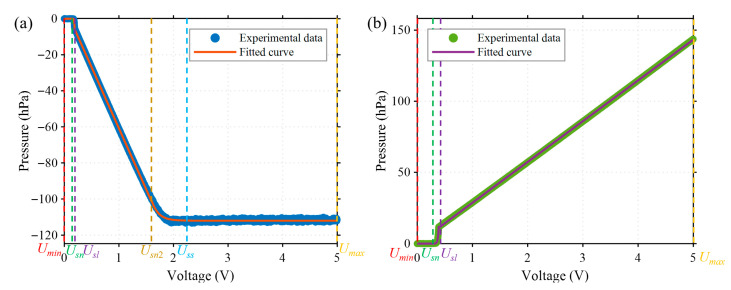
Pressure–voltage characteristics of the electronic pressure controllers (EPCs). (**a**) Negative EPC, showing a saturation region when the input voltage exceeds the threshold $U_{ss}$. (**b**) Positive EPC, which exhibits an approximately linear relationship when the input voltage exceeds *U_sl_* without a clear saturation zone.

Generally, the relationship between the output pressure and the control voltage of an electronic pressure controller (EPC) is approximately linear within the main working range. However, nonlinearity occurs in the boundary regions, including the initial low-voltage range and the saturation region at high pressures. To ensure the accuracy of the system model, the pressure–voltage characteristic was recalibrated before each startup of the pressure controller. Specifically, the input voltage of both positive and negative EPCs is swept from 0 V to 5 V with a resolution of 0.001 V, and corresponding steady-state output pressures are recorded using a pressure sensor. The resulting pressure–voltage profiles are shown in Figure 4.

The positive and negative EPCs exhibit distinct pressure–voltage characteristics (see Figure 4). Based on their response to input voltage, the relationships can be classified into two types according to whether saturation occurs in the output: saturated ($P_{s}$) and unsaturated ($P_{us}$). The negative EPC follows the saturated type, with its pressure–voltage relationship described by Equation (1):

(1) $$P_{s}\left(U\right)=\left\{\begin{array}{cc} \begin{array}{c} 0\\ a_{s}e^{b_{s}\left(U-U_{n}\right)} \end{array} & \begin{array}{c} U_{min}\leq U<U_{sn}\\ U_{sn}\leq U<U_{sl} \end{array}\\ \begin{array}{c} c_{s}U+d_{s}\\ e_{s}e^{f_{s}\left(U-U_{n2}\right)}+P_{s0} \end{array} & \begin{array}{c} U_{sl}\leq U<U_{sn2}\\ U_{sn2}\leq U<U_{max} \end{array} \end{array}\right.$$

where $U_{sn}$ is the boundary point control voltage between 0 and the nonlinear region, $U_{sl}$ is the boundary point between the nonlinear and linear regions, $U_{sn2}$ is the boundary between the linear region and the saturation region, $a_{s}$, $b_{s}$, $c_{s}$, $d_{s}$, $e_{s}$ and $f_{s}$ are the parameters to be identified. $U_{min}$ and $U_{max}$ are the minimum and maximum values of the control voltage.

As for positive EPC, the voltage-pressure relationship exhibiting non-saturation characteristics is given by Equation (2):

(2) $$P_{us}\left(U\right)=\left\{\begin{array}{cc} 0 & U_{min}\leq U<U_{un}\\ a_{u}e^{b_{u}\left(U-U_{n}\right)} & U_{un}\leq U<U_{ul}\\ c_{u}U+d_{u} & U_{ul}\leq U<U_{max} \end{array}\right.$$

where $U_{un}$ is the boundary point control voltage between 0 and the nonlinear region, $U_{ul}$ is the boundary point between the nonlinear and linear regions, and $a_{u}$, $b_{u}$, $c_{u}$ and $d_{u}$ are the parameters to be identified.

After determining the dead zone, nonlinear region, and linear region boundaries, separate fittings are performed to identify the unknown parameters. These identified parameters were then used to construct the control-oriented model, providing the basis for subsequent closed-loop pressure regulation.

### 2.3. Closed-Loop Pressure Regulator Controller Design

In order to achieve precise outlet pressure control, a closed-loop control is designed with a deviation e defined as

(3) $$e=P_{d}-P$$

where $P_{d}$ is the desired outlet pressure and P is the current pressure value.

The standard continuous time PID control law $u_{pid}$ is

(4) $$\left\{\begin{array}{c} u_{spid}=K_{ps}e+K_{is}\int_{0}^{t}e\left(\tau \right)d\tau +K_{ds}\frac{de\left(t\right)}{dt}\\ u_{uspid}=K_{pus}e+K_{ius}\int_{0}^{t}e\left(\tau \right)d\tau +K_{dus}\frac{de\left(t\right)}{dt} \end{array}\right.$$

where $K_{ps}$, $K_{is}$ and $K_{ds}$ represent the proportional, integral, and derivative coefficients of the EPC with saturated characteristics, respectively. $K_{pus}$, $K_{ius}$ and $K_{dus}$ represent the proportional, integral, and derivative coefficients of the EPC with unsaturated characteristics, respectively.

To improve the dynamic response of the system, a feedforward control strategy was implemented. Specifically, the theoretical control inputs $U_{usff}$ and $U_{sff}$, corresponding to the saturated and unsaturated modes, respectively, were computed from the inverse model of the EPC to achieve the desired output pressure $P_{d}$. For the unsaturated mode, the feedforward input is given as

(5) $$U_{usff}=\left\{\begin{array}{cc} U_{un} & P_{d}=0\\ U_{n}+\frac{1}{b_{u}}\ln\left(\frac{P_{d}}{a_{u}}\right) & 0<P_{d}\leq P_{ul}\\ \frac{P_{d}-d_{u}}{c_{u}} & P_{d}>P_{ul} \end{array}\right.$$

where $P_{ul}=a_{u}e^{b_{u}\left(U_{ul}-U_{un}\right)}$.

The saturated mode $U_{sff}$ is as follows:

(6) $$U_{sff}=\left\{\begin{array}{cc} U_{sn} & P_{d}=0\\ U_{sn}+\frac{1}{b_{s}}\ln\left(\frac{P_{d}}{a_{s}}\right) & 0<P_{d}\leq P_{sl}\\ \frac{P_{d}-d_{s}}{c_{s}} & P_{sl}<P_{d}\leq P_{sn2}\\ U_{sn2}+\frac{1}{f_{s}}\ln\left(\frac{P_{d}-P_{s0}}{e_{s}}\right) & P_{sn2}<P_{d}\leq P_{ss}\\ U_{max} & P_{d}>P_{ss} \end{array}\right.$$

where

(7) $$\left\{\begin{array}{c} P_{ul}=a_{u}e^{b_{u}\left(U_{ul}-U_{un}\right)}\\ P_{sn2}=c_{s}U_{sn2}+d_{s}\\ P_{ss}=e_{s}e^{f_{s}}\left(U_{ss}-U_{sn2}\right) \end{array}\right.$$

The final system control rate u can be defined as

(8) $$u=\left\{\begin{array}{cc} U_{uspid}+U_{usff\_actual} & P_{d}>0\\ U_{spid}+U_{sff\_actual} & P_{d}<0 \end{array}\right.$$

where $U_{usff\_actual}$ and $U_{sff\_actual}$ is the actual output control voltage of feedforward controller, which can be expressed by

(9) $$U_{usff\_actual}=\left\{\begin{array}{cc} U_{usff} & \left|P-P_{d}\right|>\varepsilon \\ U_{usff\_last\_stable} & \left|P-P_{d}\right|\leq \varepsilon \end{array}\right.$$

(10) $$U_{sff\_actual}=\left\{\begin{array}{cc} U_{sff} & \left|P-P_{d}\right|>\varepsilon \\ U_{sff\_last\_stable} & \left|P-P_{d}\right|\leq \varepsilon \end{array}\right.$$

where $U_{usff\_last\_stable}$ and $U_{sff\_last\_stable}$ is the output control voltage after the last system stabilization. ε is the critical threshold of feedforward control.

**Figure 5:**
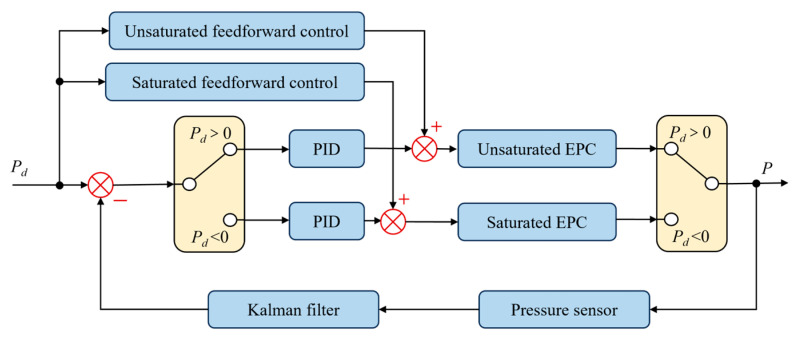
System control block diagram.

The system control block diagram is shown in Figure 5, comprising anti-integral saturation feedforward PID control and Kalman filtering for processing barometric pressure sensor data.

### 2.4. Flow Measurement Using Droplet Volume Analysis

Due to the structural limitation, existing flowmeters [26,27] cannot be directly integrated at the micropipette tip opening to measure the outlet flow rate. To address this challenge, we propose a simple and practical measurement method based on monitoring the growth of liquid droplets in silicone oil, which enables indirect yet accurate quantification of the flow rate. It is noted that the silicone oil in this work was not intended to simulate the biological environment, but forming a local liquid droplet allows accurate visualization and quantitative measurement of the outlet flow rate by tracking the change in droplet volume over time. The formed liquid droplet is utilized to simulate the real extracellular fluid environment. In actual applications, the injected fluid is a fluorescent dye dissolved in artificial cerebrospinal fluid (aCSF), whose viscosity is close to that of the real extracellular environment. The calibration process in silicone oil does not change the rheological properties of the injected liquid itself, since the environment surrounding micropipette tip is still fluorescent solution. While the previous report [7] only provided limited experimental details, the present study describes the measurement principle and experimental implementation in detail.

**Figure 6:**
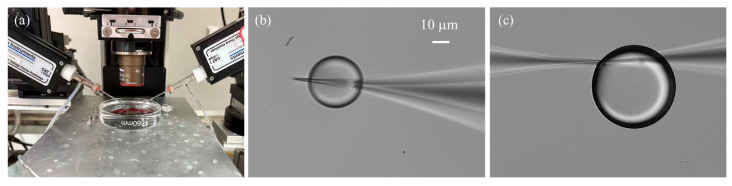
Droplet generation and stabilization. (**a**) Experimental setup for droplet generation. (**b**) Unstable growth of the droplet climbing along the outer wall of the micropipette. (**c**) Immobilization of the droplet achieved by positioning two micropipettes in close proximity.

**Figure 7:**
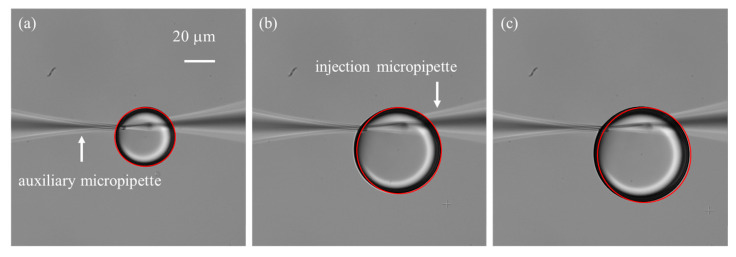
Time-lapse of droplet growth at an injection pressure of 60 hPa, recorded with an interval of 20 frames. Red outlines represent droplet boundaries detected using the Hough circle detection transform. (**a**) Initial frame. (**b**) Image at the 20th frame. (**c**) Image at the 40th frame.

In this study, silicone oil (Sigma-Aldrich, St. Louis, MO, USA) was used as the calibration medium. Its physical properties at 25 °C are as follows: viscosity = 9.7 Pa·s, density = 0.97 g·cm^−3^, and surface tension = 20.6 mN·m^−1^.The silicone oil is placed into a Petri dish with a diameter of 5 cm, illustrated in Figure 6a. The injection micropipette is carefully positioned within silicone oil layer, leaving sufficient clearance from both the Petri dish bottom and the oil–air interface to allow unobstructed droplet growth and to avoid interfacial disturbances. We often select the work focal plane as the middle of the silicone oil layer thickness. The growth of the injected droplet in silicone oil is continuously recorded using the CCD camera and a supplementary video named Video S1: Droplet Growth.mp4 is provided to illustrate the droplet growth process in real time. Time-lapse image sequences are acquired at a frame rate of 2 fps. Image sequences of droplet growth are first processed using the Hough circle transform to detect the droplet boundary. The radius r of the droplet can be calculated, as shown in Figure 7. The microdroplet in the silicone oil can be well approximately spherical due to the dominance of surface tension over gravity [28]. Thus, the volume can be calculated as follows:

(11) $$V=\frac{4}{3}\pi r^{3}$$

The outlet flow rate can be obtained as the temporal derivative of the droplet volume:

(12) $$Q(t)=\dot{V}(t)$$

Direct point-to-point differentiation was avoided due to its sensitivity to imaging noise. Instead, least-squares fitting was applied to the volume–time data, and the slope of the fitted curve was taken as the flow rate (detailed results are provided in Section 3.2 and Section 3.3).

One important consideration is the small opening size of the micropipette, typically only a few micrometers, which leads to a significant surface tension barrier between the liquid solution and the silicone oil [29]. As a result, a relatively large initial injection pressure is required to initiate droplet formation. In practice, two common situations are often encountered: (1) the applied initial pressure is insufficient, preventing the formation of microdroplets; and (2) the initial pressure is excessive, producing oversized droplets whose growth is difficult to control. To address this challenge, we employed an auxiliary micropipette with a larger opening size (~3 μm) to generate an initial droplet adjacent to the injection micropipette tip, thereby immersing the tip into the droplet. The two micropipettes were carefully positioned with their tips placed within 1 µm of each other and aligned in the same focal plane. Experiment results demonstrated that the auxiliary micropipette not only facilitated overcoming the initial surface tension barrier but also stabilized the subsequently formed microdroplet. This stabilization is critical because the injection micropipette tip is both hydrophilic and cone-shaped, which tends to cause the droplet to climb along the outer wall (Figure 6b), disturbing its continuous growth and affecting flow rate measurements. By introducing the auxiliary micropipette, the forces acting on the droplet were balanced (Figure 6c), effectively immobilizing it and ensuring reliable flow quantification.

### 2.5. Sample Preparation

The injection micropipette in this study is pulled by the specialized puller (P-97, Sutter Instrument, Novato, CA, USA) from standard glass capillary (BF150-86-10, Sutter Instrument, Novato, CA, USA) with outer diameter of 1.5 mm and inner diameter of 0.86 mm. The pull program used in this study is set as follows: heat = 540, pull = 0, velocity = 30, and time = 250. It should be noted that the heat parameter must be individually calibrated for each puller, as variations in filament characteristics and equipment condition can significantly affect the actual heating efficiency. The values reported here therefore serve only as a reference. In addition, these parameters are dimensionless, instrument-specific settings rather than physical quantities with SI units. The size of its opening is ~1 µm, measured by scanning electron microscope (SEM). The auxiliary micropipette is fabricated from the same type of glass capillary as the injection micropipette. In the puller program, the heat parameter is set approximately 50 units higher than that used for the injection micropipette in order to produce a longer and thinner tip, facilitating further fabrication for wider tip opening. Then, the pipette is further processed by microforge trimming (MF-900, Narishige Co., Ltd., Tokyo, Japan), to achieve a final opening diameter of 10~20 µm.

The micropipette is backfilled with 20 µL fluorescent solution with concentration of 50 µmol/L. Because the experimental validation of this study is mainly carried out in the context of patch clamp recordings, the internal micropipette solution is prepared with a composition similar to the cytoplasmic milieu, thereby ensuring physiological relevance and compatibility with neuronal recordings. The internal solution contains (in mM): 150 K-gluconate, 10 KCl, 10 HEPES, 0.25 EGTA, 5 Mg-ATP (pH 7.2, 280 mOsm). This formulation allowed stable electrode–cell interactions during in situ electrophysiological measurements. For applications outside the patch clamp context, however, the requirement is less stringent; it is generally sufficient to use a simple conductive solution to ensure reliable ionic current flow within the micropipette.

Although the ultimate goal of this study is to enable in vivo applications of precise micropipette flow control, the current validation experiments were conducted in acute mouse brain slices. This choice was made due to experimental limitations and the need for a more stable and reproducible preparation, while still maintaining key physiological properties of in vivo environment. Acute brain slices were prepared from C57BL/6 mice (5–8 weeks). Animals were deeply anesthetized with isoflurane and rapidly decapitated in accordance with institutional animal care and use guidelines. The brain was quickly removed and immersed in ice-cold, oxygenated cutting solution containing (in mM): 125 NaCl, 2.5 KCl, 1 MgCl_2_, 2 CaCl_2_, 1.25 NaH_2_PO_4_, 25 NaCO_3_, 25 C_6_H_12_O_6_ (pH 7.4, bubbled with 95% O_2_/5% CO_2_). Coronal slices (300 μm thickness) containing the cortex were cut using a vibratome (7000 smz-2, Campden Instruments Ltd, Loughborough, UK). Slices were then transferred to a custom-designed holding chamber filled with oxygenated artificial cerebrospinal fluid (ACSF) composed of the same composition as the cutting solution containing. The slices were incubated at 37 °C for 30 min for recovery and subsequently maintained at room temperature until use. During experiments, individual slices were transferred to a recording chamber and continuously superfused with oxygenated ACSF at 32–34 °C.

All animal experiments were approved by the Experimental Animal Ethics Committee, Nankai University and conducted in accordance with the Animal Management Rules of the Ministry of Health of the People’s Republic of China.

## 3. Results

### 3.1. Pressure Control Results

For the model parameter fitting section of the EPC, $a_{s}\;$= −5.73, $b_{s}$ = 171.62, $c_{s}$ = −67.79, $d_{s}$ = 6.97, $e_{s}$ = 0.40, $f_{s}$ = −7.94, $P_{s0\;}$= 112.64, $U_{sn}\;$= 0.15, $U_{sl}\;$= 0.19, $U_{sn2}$ = 1.60, $U_{min}$ = 0, $U_{max}$ = 5.00, RMES (negative EPC) = 0.470 hPa, $a_{u}$ = 0.74, $b_{u}$ = 70.87, $c_{u}$ = 28.68, $d_{u}$ = −0.043, $U_{un}$ = 0.25, $U_{ul}\;$= 0.39, RMES (positive EPC) = 0.217 hPa, demonstrating its excellent fitting characteristics. In the control section, after extensive experimental adjustments, $k_{ps}$ = −2 × 10^−4^, $k_{is}$ = −2 × 10^−4^, $k_{ds}$ = −1 × 10^−5^, $k_{pus}$ = 1 × 10^−4^, $k_{ius}$ = 5 × 10^−4^, $k_{dus}$ = 1 × 10^−5^. To mitigate the impact of model identification errors on the feedforward controller, we set *ε* = 1 hPa.

**Figure 8:**
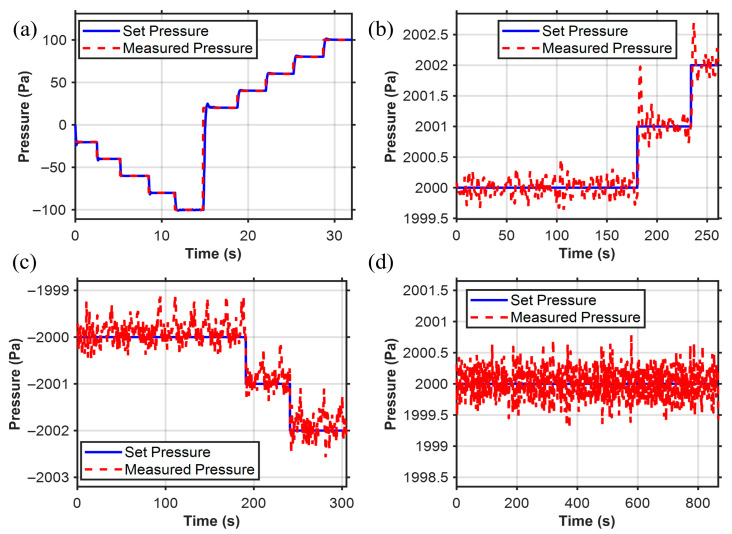
Pressure controller results. (**a**) Pressure sensor operates within the range of pressure switching (−100 to 100 hPa). (**b**) Pa-level control resolution within the positive pressure range. (**c**) Pa-level control resolution within the negative pressure range. (**d**) Long-term working stability (error is less than 0.7 Pa).

We set the desired pressure *P_d_* within the controllable pressure range of −100 hPa to 100 hPa. The pressure adjustment time is 0.5 s, with a steady-state error below 1 Pa, enabling pressure control at the Pa level as shown in Figure 8a. This demonstrates the wide range of the pressure controller. In the research of the pressure controller, we verified that it has the ability to regulate pressure with a resolution of 1 Pa level both in positive pressure shown in Figure 8b and negative pressure shown in Figure 8c. We also conducted a long-term (~13 min) pneumatic pressure stability test as shown in Figure 8d, and the results indicated that the pneumatic pressure could be maintained within ±0.7 Pa, with Pa-level stable pneumatic pressure control.

### 3.2. Calibration of the Relationship Between Injection Pressure and Outlet Flow Rate

**Figure 9:**
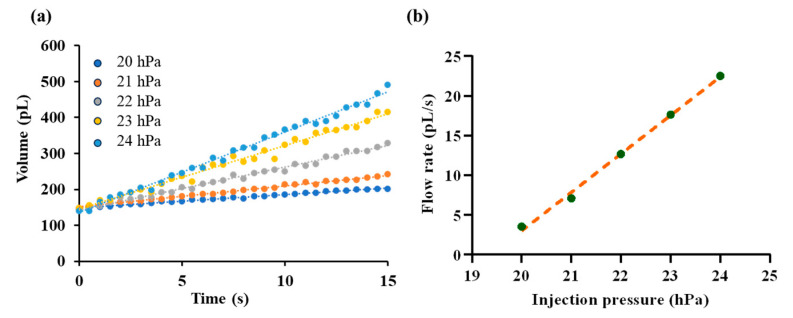
Calibration of the pressure–flow relationship. (**a**) Time-dependent growth of droplet volume under different injection pressures, ranging from 20 hPa to 24 hPa. (**b**) Fitted linear relationship between injection pressure and the outlet flow rate.

In this section, fluorescent solution was injected into silicone oil under various injection pressure ranging from 20 hPa to 24 hPa with resolution of 1 hPa. The injected droplet volume is recorded over time as shown in Figure 9a and corresponding outlet flow rate is calculated as described in Section 2.4. Steady-state flow rates are extracted for each pressure level. By fitting the paired data of injection pressure and outlet flow rate, a linear relationship was obtained, as illustrated in Figure 9b. The fitting equation is given by

(13) $$Q=\alpha (P-P_{h})$$

where $P_{h}$ denotes the hydrostatic pressure of the silicone oil. In the silicone oil, $P_{h}$ can be expressed as

(14) $$P_{h}=\frac{2\gamma }{r}$$

in which γ is the interfacial tension between the fluorescent solution and silicone oil, and r is the radius of the microdroplet. To eliminate this effect on the calibration of the pressure–flow relationship, we carefully maintained the same initial droplet radius with radius of ~35 µm in all tests. The fitted slope was $\alpha =4.846\;pL\cdot s^{-1}\cdot {hPa}^{-1},$ with a coefficient of determination $R^{2}=0.996$, indicating a good agreement with the linear model.

The linear relationship is consistent with the theoretical prediction of laminar flow through a capillary at low Reynolds number. Under such conditions, viscous forces dominate while inertial effects are negligible, resulting in a direct proportionality between pressure drop and volumetric flow rate as described by Poiseuille’s law [30], given as

(15) $$Q=\frac{\pi ∆PR_{o}^{4}}{8\mu L}\cdot \frac{3\lambda^{3}}{1+\lambda +\lambda^{3}},\;\lambda =\frac{R_{L}}{R_{o}}$$

where ∆P is the applied pressure drop, μ is the dynamic viscosity of the injected solution, L is the effective length of the liquid column, and $R_{o}$ and $R_{L}$ are the inner radius of the micropipette tip and liquid meniscus, respectively. Accordingly, the injected volume follows V=Qt, showing a linear dependence on the injection time t, while Q increases linearly with ∆P. Since the initial Laplace pressure $P_{h}$ was kept constant for all calibration tests, the effective pressure drop $\left(P-P_{h}\right)$ varied linearly with the applied injection pressure P. As a result, the observed linear dependence of flow rate on P is equivalent to a linear relationship between the flow rate and the applied pressure, further confirming the consistency of the experimental results with theoretical expectations.

The identified relationship provides a quantitative calibration between injection pressure and micropipette outlet flow, which forms the basis for subsequent closed-loop flow regulation experiments.

### 3.3. Precise Micropipette Flow Rate Control Results

Based on the calibrated relationship between injection pressure and outlet flow rate, the proposed closed-loop pressure regulator was employed to achieve specified flow rate control. In this section, we evaluated system performance by setting three target flow rates: 5 pL/s, 15 pL/s, and 25 pL/s. The results obtained with the proposed system were compared with those from manual operation using a pneumatic microinjector (IM-11-2, NARISHIGE, Tokyo, Japan).

**Figure 10:**
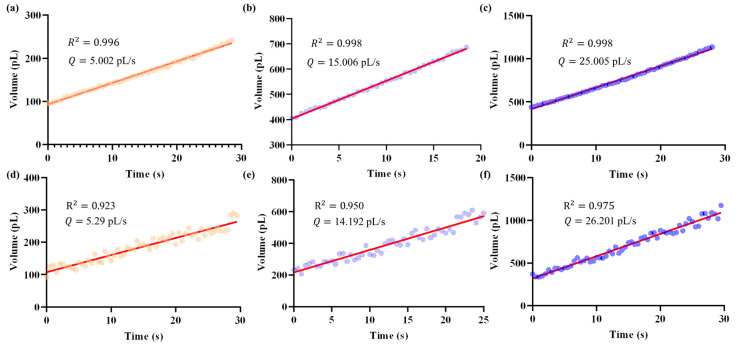
Temporal evolution of droplet volume (represented by dots) and the corresponding micropipette outlet flow rates obtained as slopes from least-squares fitting (red line), under different target values. Data were recorded at a sampling rate of 2 Hz. Panels (**a**–**c**) show the results achieved by the proposed closed-loop system, while panels (**d**–**f**) correspond to manual operation. Using this method, the closed-loop regulator achieved mean flow rates of 5.002 pL/s, 15.006 pL/s, and 25.005 pL/s for the three target values. In contrast, manual operation resulted in less accurate and more variable outcomes, with mean flow rates of 5.29 pL/s, 14.192 pL/s, and 26.201 pL/s, respectively.

Figure 10 presents representative results, showing the temporal evolution of droplet volumes and the fitted results under different target values. The closed-loop system consistently regulated the outlet flow close to the specified targets (Figure 10a–c), whereas manual operation exhibited larger deviations and poor reproducibility (Figure 10d–f). Notably, the flow rate traces reveal that the proposed system maintained a smooth and low-noise profile, while manual regulation produced pronounced fluctuations.

Quantitatively, the outlet flow rate was obtained as the slope from least-squares fitting of the volume change over time. Using this method, the closed-loop regulator achieved mean flow rates of 5.002 pL/s, 15.006 pL/s, and 25.005 pL/s for the three targets, with corresponding coefficients of determination of 0.996, 0.998, and 0.998, respectively. In contrast, manual operation resulted in less accurate and more variable outcomes: 5.29 pL/s, 14.192 pL/s, and 26.201 pL/s, with lower R^2^ values of 0.923, 0.950, and 0.975, respectively.

Importantly, the closed-loop system achieved a flow control error less than 10 fL/s, highlighting its capability to achieve precise and reproducible regulation of micropipette outlet flow rates at the sub-picoliter per second scale. This level of stability and resolution is critical for reliable fluid injection in in vivo micromanipulations. This comparison highlights the advantages of the pressure regulator over manual operation, particularly in terms of both response speed and control accuracy, thereby demonstrating its potential for reliable and reproducible micropipette flow regulation in practical applications.

### 3.4. Closed-Loop Flow Rate Control for Fluorescence Imaging in Brain Slice

Two-photon fluorescence imaging enables visualization of cellular structures deep within living tissues. In this technique, a focused near-infrared laser excites fluorescent molecules only at the focal point through simultaneous absorption of two photons, thereby minimizing photodamage and out-of-focus background. When the fluorescent dye is injected into the extracellular space, it illuminates the surrounding microenvironment while excluding the intracellular region. As a result, cells appear as dark silhouettes against a bright fluorescent background, allowing clear visualization of their morphology and spatial distribution in vivo. Stable fluorescence imaging is essential for achieving high-quality cellular visualization in vivo, which fundamentally relies on maintaining stable fluorescence injection.

To validate the effectiveness of the proposed outlet flow control system, fluorescence injection experiments were conducted in brain-slices environments. In this experiment, direct measurement of the flow rate was not feasible due to the microscale injection volume and the complex biological medium. Therefore, the fluorescence intensity surrounding the micropipette tip was employed as an indirect indicator of the solution flow rate. This is reasonable because the observed brightness arises from the dynamic balance between injection-driven convection and molecular diffusion. When the outlet flow rate increases, more fluorescent molecules are delivered per unit time, leading to a higher local concentration near the micropipette tip and thus stronger fluorescence emission. Conversely, a reduction in flow rate lowers the molecular supply and results in weaker fluorescence. Therefore, variations in fluorescence intensity directly reflect changes in flow rate, providing a reliable basis for evaluating the stability of flow control.

**Figure 11:**
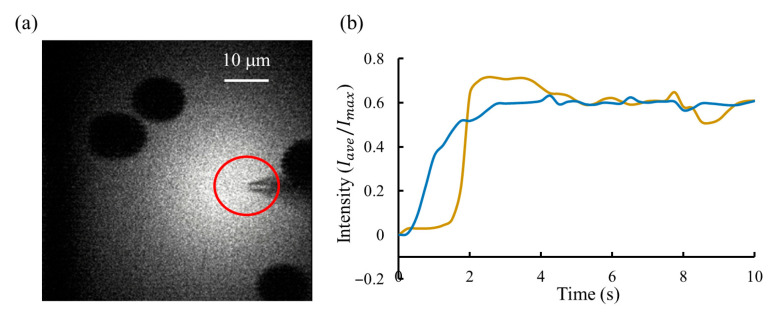
Fluorescent lighting results. (**a**) Two-photon fluorescence imaging of brain-slice tissue during continuous injection of the fluorescent solution. The shadowed regions correspond to cell bodies, which appear dark because the fluorescent dye remains in the extracellular space and does not enter the cells. The red-circled area marks the region of interest (ROI) selected for fluorescence intensity analysis. (**b**) Intensity regulation results comparison between the proposed system (blue line) and manual operation (yellow line). A stable fluorescence trace indicates consistent flow regulation, demonstrating the system’s stable control performance.

In the experiments, the region of interest (ROI) was defined as a circular area of 20 μm in diameter surrounding the micropipette tip, as shown in Figure 11a. The intensity value was defined as the ratio between the average intensity of the ROI and the brightest pixel intensity in the image, with the setpoint selected at 0.6. Two performance metrics were used: (1) settling time, defined as the interval between initiating the injection command from the GUI and the time at which fluorescence intensity entered and remained within the steady-state band around the setpoint; and (2) steady-state accuracy (SSA), defined as the root-mean-square (RMS) deviations of the steady-state normalized intensity from the setpoint, as given by follows:

(16) $$SSA=\sqrt{\frac{1}{N}\sum_{i=1}^{N}{\left(I_{i}-I_{set}\right)}^{2}}$$

where $I_{i}$ is the normalized fluorescence intensity at the i-th sampling point in the steady-state, $I_{set}$ is the setpoint intensity, and N is the number of samples considered within the steady-state interval.

Figure 11b compares the regulation results between these two approaches: the proposed pressure regulator and manual operation. The proposed system achieved the fast response, with a settling time of less than 3 s, compared to more than 4 s for manual operation. In addition, it provided the highest steady-state accuracy, with a fluorescence intensity SSA of 1.3%, versus 4% for manual operation, demonstrating a stable injection flow rate of fluorescent dye. At the same time, we recorded the micropipette resistance, which indicates the degree of contamination at the micropipette tip. Results showed that the clogging rate under dynamic injection control (30%, 3 micropipettes/10 micropipettes) was reduced by about half compared with that during manual operation (60%, 6 micropipettes/10 micropipettes).

Taken together with Section 3.2 and Section 3.3, these results confirm that the system not only enables precise flow regulation under controlled conditions, but also maintains stable performance in physiologically relevant environments. 

## 4. Discussion

For the calibrated pressure–flow relationship, it should be emphasized that the hydrostatic pressure of the surrounding environment directly affects the actual micropipette outlet flow rate. In Section 3.4, when experiments were performed in brain slices, the local hydrostatic pressure was unknown, making the absolute outlet flow rate difficult to determine. Nevertheless, the proposed system was able to regulate the injection process such that the flow remained stable over time.

The proposed system demonstrated stable and precise flow-rate regulation, as evidenced by the fluorescence intensity fluctuation of only 1.3% during in brain slices environment validation. This low variation is reasonable and reflects the stable balance between injection and diffusion of fluorescent molecules achieved under controlled conditions. Specifically, the brain-slice environment used in the validation experiments is relatively homogeneous, maintaining stable physical parameters such as temperature, pressure, and extracellular viscosity. Under such quasi-stationary conditions, once the injection–diffusion equilibrium is established, large temporal fluctuations in local fluorescence intensity are unlikely to occur. However, in more heterogeneous biological tissues—such as vascular or fibrous regions—local variations in permeability and viscosity may alter the diffusion dynamics and result in higher fluorescence fluctuations. Therefore, while the current results verify the system’s capability for stable flow regulation under homogeneous conditions, future studies will further investigate its performance in complex in vivo environments with spatial heterogeneity.

The observed linear relationship between applied pressure and outlet flow rate is valid under laminar flow conditions and when the micropipette behaves as a rigid channel. In this regime, viscous force remains constant, leading to a proportional pressure–flow response as predicted by Poiseuille’s law. However, this linearity may deviate when the pressure gradient is sufficiently large to cause wall deformation, or when the working fluids or external media exhibit non-Newtonian behavior. In compliant or heterogeneous environments, variations in local viscosity or interfacial tension could also induce nonlinear pressure–flow characteristics. In our experiments, the use of a glass micropipette and Newtonian solution ensured that such nonlinear effects were negligible, supporting the validity of the linear approximation.

The potential influence of gas compressibility on control stability was also considered. In principle, gas in a long pneumatic line can introduce a phase delay between the control signal and the actual outlet pressure. However, in our system, this effect is negligible because the baseline pressure (on the order of kPa) is sufficiently high to overcome the capillary force at the micropipette tip (~1 µm in diameter). Under this pre-compressed condition, subsequent pressure adjustments of only tens of pascals cause minimal volume variation, resulting in an insignificant phase delay. Nevertheless, for systems employing larger pipette openings or lower baseline pressures, the compressibility effect could become more pronounced and should be carefully accounted for in future designs.

## 5. Conclusions

In this study, we proposed and validated a closed-loop pressure regulation system for precise control of micropipette outlet flow for fluorescence imaging in in vivo micromanipulations. The system integrates a multi-channel pressure regulation unit with 1 Pa resolution and was modeled as a first-order plus dead-time process to capture the compressibility and compliance of the injection structure. By employing a droplet-based calibration method, we establish a quantitative and highly linear pressure–flow relationship, enabling accurate mapping between injection pressure and outlet flow rate.

Compared with existing microinjection and microfluidic control systems, the proposed approach demonstrates distinct advantages in both control accuracy and stability. Commercial microinjection controllers (e.g., FemtoJet) lack quantitative pressure–flow calibration and cannot precisely control injected volume, while syringe-based or microfluidic systems typically achieve only microliter-per-minute resolution and are limited to in vitro applications. In contrast, the present system achieves femtoliter-per-second flow-rate prediction with an error below 10 fL/s and maintains fluorescence intensity fluctuations as low as 1.3% in brain-slice environment validation. Validation experiments confirmed that the regulator can achieve target outlet flows with the control accuracy of 10 pL/s, while applications in patch clamp operations demonstrated stable fluorescent dye solution injection with fluorescence fluctuations around 1.3%, and less than 4% for manual operation.

These results confirm that the proposed system provides stable, precise, and reproducible flow regulation under physiologically relevant conditions, offering a practical and scalable tool for in vivo microinjection, neural imaging, and other microscale biofluidic operations.

## Data Availability

The original contributions presented in this study are included in the article/Supplementary Material. Further inquiries can be directed to the corresponding author.

## Supplementary Materials

The following supporting information can be downloaded at: https://www.mdpi.com/article/10.3390/s25216647/s1, Video S1: Droplet Growth.

## References

[1] Davidson S., Truong H., Nakagawa Y., Giesler G.J. (2010). A Microinjection Technique for Targeting Regions of Embryonic and Neonatal Mouse Brain In Vivo. Brain Res..

[2] Zhang Y., Yu L.-C. (2008). Microinjection as a Tool of Mechanical Delivery. Curr. Opin. Biotechnol..

[3] Stewart M.P., Sharei A., Ding X., Sahay G., Langer R., Jensen K.F. (2016). In Vitro and Ex Vivo Strategies for Intracellular Delivery. Nature.

[4] Dembitskaya Y., Boyce A.K., Idziak A., Pourkhalili Langeroudi A., Arizono M., Girard J., Le Bourdellès G., Ducros M., Sato-Fitoussi M., Ochoa de Amezaga A. (2023). Shadow Imaging for Panoptical Visualization of Brain Tissue In Vivo. Nat. Commun..

[5] Kitamura K. (2012). Two-Photon Targeted Patch-Clamp Recordings In Vivo. Patch Clamp Techniques: From Beginning to Advanced Protocols.

[6] Quek Y.J., Tay A. (2024). Nanoscale Methods for Longitudinal Extraction of Intracellular Contents. Adv. Mater..

[7] Li R., Li J., Li Z., Qiu J., Hu B., Li M., Sun M., Zhao X., Zhao Q. (2025). Robotic Fluorescent Lighting Method Based on Dynamic Fluorescence Imaging Modeling for In Vivo Cell Manipulation. IEEE Trans. Autom. Sci. Eng..

[8] Liu F., Wu D., Wu X., Chen K. (2015). Analyses of the Cell Mechanical Damage during Microinjection. Soft Matter.

[9] Ilerioluwa L., Zhang H., Bai C., Wang C., Zheng Y., Wang X., Sun Y., Guobin L. (2022). A Multi-Parameter Microfluidic Particle Sensor Based on Permalloy for High Sensitivity. IEEE Trans. Instrum. Meas..

[10] Chow Y.T., Chen S., Wang R., Liu C., Kong C., Li R.A., Cheng S.H., Sun D. (2016). Single Cell Transfection through Precise Microinjection with Quantitatively Controlled Injection Volumes. Sci. Rep..

[11] Zhuang S., Lei D., Yu X., Tong M., Lin W., Rodriguez-Andina J.J., Shi Y., Gao H. (2024). Microinjection in Biomedical Applications: An Effortless Autonomous Omnidirectional Microinjection System. IEEE Ind. Electron. Mag..

[12] Kallio P., Kuncova-Kallio J. (2006). Capillary Pressure Microinjection of Living Adherent Cells: Challenges in Automation. J. Micromechatron..

[13] Yamada H., Yaginuma T., Mase K., Niimi T. (2025). Egg Microinjection for the Silkworm Bombyx Mori. Bio Protoc..

[14] Eroglu A., Lawitts J.A., Toner M., Toth T.L. (2003). Quantitative Microinjection of Trehalose into Mouse Oocytes and Zygotes, and Its Effect on Development. Cryobiology.

[15] Kern R., Stobrawa S. Step-by-Step Guide: Microinjection of Adherent Cells with the Eppendorf InjectMan^®^ 4 and FemtoJet^®^ 4. *Short protocol*. https://www.eppendorf.com/product-media/doc/en/825836/Cell-Technology_Protocol_047_InjectMan-4-FemtoJet-4_Step-Step-Guide-Microinjection-Adherent-Cells-Eppendorf-InjectMan-4-FemtoJet-4.pdf.

[16] Fleming S.D. (2021). Micromanipulation, Microscopes Micro-Injection and Systems for ICSI. Manual of Intracytoplasmic Sperm Injection in Human Assisted Reproduction: With Other Advanced Micromanipulation Techniques to Edit the Genetic and Cytoplasmic Content of the Oocyte.

[17] Sugimoto Y., Naniwa K., Aonuma H., Osuka K. (2020). Microinjection Support System for Small Biological Subjects. HardwareX.

[18] Gonzalez G.I.V., Kgwarae P.O., Schultz S.R. (2023). Two-Photon Targeted, Quad Whole-Cell Patch-Clamping Robot. Proceedings of the 2023 11th International IEEE/EMBS Conference on Neural Engineering (NER).

[19] Li Z.-Y., Liu Y.-Y., Li Y.-J., Wang W., Song Y., Zhang J., Tian H. (2021). High-Preservation Single-Cell Operation through a Photo-responsive Hydrogel-Nanopipette System. Angew. Chem. Int. Ed..

[20] Xu S., Hu B., Liu R., Zhao X., Sun M. (2024). Liquid-Driven Microinjection System for Precise Fundus Injection. Sensors.

[21] Yuan S., Zhou M., Liu X., Jiang B. (2023). Effect of Pressure-Driven Flow on Electroosmotic Flow and Electrokinetic Mass Transport in Microchannels. Int. J. Heat Mass Transf..

[22] de Graaf M.N., Vivas A., van der Meer A.D., Mummery C.L., Orlova V.V. (2022). Pressure-Driven Perfusion System to Control, Multiplex and Recirculate Cell Culture Medium for Organs-on-Chips. Micromachines.

[23] Lake J.R., Heyde K.C., Ruder W.C. (2017). Low-Cost Feedback-Controlled Syringe Pressure Pumps for Microfluidics Applications. PLoS ONE.

[24] Zhang Y., Tseng T.-M., Schlichtmann U. (2021). Portable All-in-One Automated Microfluidic System (PAMICON) with 3D-Printed Chip Using Novel Fluid Control Mechanism. Sci. Rep..

[25] Hettiarachchi S., Melroy G., Mudugamuwa A., Sampath P., Premachandra C., Amarasinghe R., Dau V. (2021). Design and Development of a Microfluidic Droplet Generator with Vision Sensing for Lab-on-a-Chip Devices. Sens. Actuators A Phys..

[26] Li M., Li Z., Li C. (2023). In-Use Measurement of Ultrasonic Flowmeter Based on Machine Learning. Measurement.

[27] Cavaniol C., César W., Descroix S., Viovy J.-L. (2022). Flowmetering for Microfluidics. Lab Chip.

[28] Harz M., Knoche M. (2001). Droplet Sizing Using Silicone Oils. Crop Prot..

[29] Guo X., Zhao A., Zhang Y., Jiang H., Tang L., Lu B., Ying Y., Zhou M. (2025). Design and Developing a Robot-Assisted Cell Batch Microinjection System for Zebrafish Embryo. Microsyst. Nanoeng..

[30] Baker L., Hieftje G., Karty J., Ray S., Saha-Shah A., Weber A. (2015). Nanopipettes: Probes for Local Sample Analysis. Chem. Sci..

